# Neonatal Perforated Appendicitis Masquerading as Necrotizing Enterocolitis

**DOI:** 10.21699/jns.v6i2.482

**Published:** 2017-04-15

**Authors:** Andrew Tumen, Pranit N Chotai, John Matthew Williams, Adrianne Myers-Webb, Jie Zhang, Ramesh Krishnan, James W Eubanks III

**Affiliations:** 1 Division of Pediatric Surgery, Le Bonehur Children's Hospital, University of Tennessee Health Science Center, Memphis, TN, USA; 2 Department of Surgery, Vanderbilt University Medical Center, Nashville, TN, USA

**Keywords:** Neonatal perforated appendicitis, Neonatal appendicitis, Necrotizing enterocolitis, Localized NEC

## Abstract

A preterm neonate underwent emergent laparotomy for presumed necrotizing enterocolitis (NEC). Intra-operatively, neonatal perforated appendicitis (NPA) was encountered. This may represent a form of NEC localized to the appendix. A high index of clinical suspicion and early laparotomy are recommended.

## CASE REPORT

An 1890-g male product of a dichorionic-diamniotic twin intrauterine pregnancy was born at 36 0/7 weeks’ gestation to a 27-year-old G2P3 mother who had received regular antenatal care. The prenatal labs and habit history were unremarkable; prenatal ultrasound demonstrated intrauterine growth restriction (IUGR). Following spontaneous onset of labor, twin-A was delivered vaginally, and our patient was then delivered by cesarean section for footling breech presentation. Apgar scores at 5 and 10 minutes were 8 and 10, respectively, with initial heart rate between 100-120 beats per minute and oxygen saturation <70% responsive to brief positive pressure ventilation. The patient was admitted to the Neonatal Intensive Care Unit (NICU) for further management and care. His initial NICU stay was uneventful aside from physiologic hyperbilirubinemia requiring phototherapy and recalcitrant hypoglycemia requiring parenteral alimentation and glucose infusion. He remained hemodynamically normal on room air through the first nine days of life. He was tolerating oral feeds on fortified breast milk and appropriately gained weight to 1960-grams. 

On the tenth day of his NICU stay he developed feeding intolerance and mild fever to 38 °C. The abdomen was moderately distended with mild diffuse tenderness to palpation and hypoactive bowel sounds in all 4 quadrants. Minimal abdominal wall erythema was noted but no overt signs of peritonitis, palpable hernias, or masses were appreciated. Abdominal radiograph demonstrated gaseous distension and pneumatosis intestinalis of bowel loops in the right lower and upper quadrants (Fig.1A). The white blood cell count was 6,000 cells/mm3 with 8% bands. C-reactive protein was 168mg/dL. Comprehensive metabolic panel was within normal range. He was made nil per oral, a nasogastric tube was placed, blood cultures were drawn, and he was started on intravenous vancomycin (15mg/kg), amikacin (15mg/kg), and a loading dose of metronidazole (15mg/kg). Repeat abdominal radiograph three hours later revealed intraperitoneal free air (Fig.1B). Patient was emergently brought to the operating room for exploratory laparotomy, washout and possible bowel resection, for a presumptive diagnosis of Bell stage III NEC. 

Intra-operative findings included viable loops of bowel with fibrinous exudates in the right lower quadrant. The appendix was found perforated at its apex, (Fig.2A), and an appendectomy was performed. No fecalith was identified. Pathologic analysis of the specimen revealed an acutely inflamed 2.5 cm blue-grey necrotic-appearing appendix open at both ends as well as mucosal necrosis with transmural acute inflammation with predominant neutrophil infiltration (Fig.2B). Postoperative recovery was uneventful. The patient was recently seen in our clinic at five months’ age, where he weighed 6300-g (below the 5th percentile). He has tolerated his diet well and had no further surgical complications.

**Figure:**
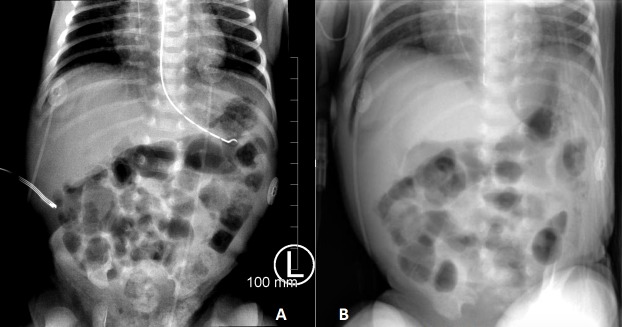
Figure 1: A) Initial plain abdominal radiograph demonstrating pneumatosis intestinalis. B) Follow-up plain abdominal radiograph demonstrating intra-peritoneal, extra-luminal gas.

**Figure:**
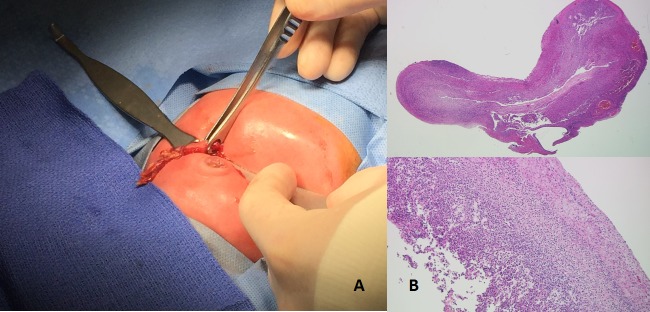
Figure 2: A) Intra-operative picture demonstrating the perforated appendix delivered through transverse supra-umbilical skin incision. B) Pathologic exam of the appendix demonstrating mucosal necrosis with transmural acute inflammation (HE, x20), and predominant neutrophil infiltration (HE, x100).

## DISCUSSION

The clinical presentation of appendicitis in the newborn is nonspecific and overlaps with that of NEC [1-8]. The most common findings are abdominal distension, tenderness, feeding intolerance, and fever [1,2]. Approximately 50% of cases occur in premature neonates and a third of cases are initially diagnosed as NEC [2]. Neonates with perforated appendicitis are usually diagnosed intra-operatively. Diagnostic delay may lead to unrecognized perforation and rapid development of abdominal sepsis. In fact, up to 85% of cases are complicated by perforation, however pneumoperitoneum is recognized in only half of perforated cases [2]. Paradoxically, perforation heralds significantly lower mortality than non-perforated cases due to timely clinical recognition, highlighting the benefit of early surgical intervention [2]. The reported mortality rate in recent years ranges from 18-28% [1,2].

Like in NEC, our preterm neonate presented during the second week of life, after initiation of enteral nutrition. Our patient also demonstrated low birth weight, IUGR, and hypoglycemia, which are established predisposing factors for NEC [6,9]. While fulminant NEC usually involves the colon and/or small bowel globally, perforated appendicitis may be the result of NEC confined, at least initially, to the appendix [4-8]. Primary appendicitis can be remarkably difficult to differentiate from isolated NEC appendicopathy, and the two cannot be histologically distinguished [4,7,10]. However, right-sided intramural gas and pneumoperitoneum in an otherwise healthy premature neonate without comorbid risk factors for appendicitis may indicate that a localized variant of NEC is responsible for appendicular perforation. We believe our 10-day-old preterm neonate without systemic co-morbidities presented with a form of NEC localized to the appendix with resultant perforation.

## Footnotes

**Source of Support:** Nil

**Conflict of Interest:**None 
